# Colliding Challenges Part 2: An Analysis of SARS-CoV-2 Infection in Patients with Extrapulmonary Tuberculosis Versus SARS-CoV-2 Infection Alone

**DOI:** 10.3390/medicina60122071

**Published:** 2024-12-16

**Authors:** Camil Mihuta, Adriana Socaci, Patricia Hogea, Emanuela Tudorache, Monica Simina Mihuta, Cristian Oancea

**Affiliations:** 1 Department of Doctoral Studies, “Victor Babes” University of Medicine and Pharmacy, 300041 Timisoara, Romania; camil.mihuta@umft.ro; 2 Clinical Hospital for Infectious Diseases and Pneumology “Dr. Victor Babes”, 300041 Timisoara, Romania; hogea.patricia@umft.ro (P.H.); emanuela.tudorache@umft.ro (E.T.); oancea@umft.ro (C.O.); 3 Department of Biology and Life Sciences, Faculty of Medicine, “Vasile Goldis” Western University of Arad, 310025 Arad, Romania; 4 Center for Research and Innovation in Precision Medicine of Respiratory Diseases (CRIPMRD), “Victor Babes” University of Medicine and Pharmacy, 300041 Timisoara, Romania; 5 Department of Pulmonology, “Victor Babes” University of Medicine and Pharmacy, 300041 Timisoara, Romania; 6 Department of Pediatrics, Faculty of Medicine, “Victor Babes” University of Medicine and Pharmacy, 300041 Timisoara, Romania; simina.mihuta@umft.ro; 7 Center of Molecular Research in Nephrology and Vascular Disease, Faculty of Medicine, “Victor Babes” University of Medicine and Pharmacy, 300041 Timisoara, Romania

**Keywords:** COVID-19 outcomes, extra-pulmonary tuberculosis, SARS-CoV-2, extra-pulmonary tuberculosis coinfection

## Abstract

*Background and Objectives*: Coinfection with SARS-CoV-2 and extrapulmonary tuberculosis (extraPTB) presents unique clinical challenges due to dual inflammatory responses and potential differences in patient profiles compared to those with SARS-CoV-2 infection alone. This study uniquely contributes to the underexplored interaction between extraPTB and SARS-CoV-2, focusing on systemic inflammation as a critical determinant of outcomes. *Materials and Methods:* This retrospective, cross-sectional study included 123 patients aged 19–91 years, hospitalized at Victor Babeș Hospital in Timișoara from March 2020 to March 2022. We compared 23 extraPTB and SARS-CoV-2 coinfected patients with 100 age-matched SARS-CoV-2-only patients. Clinical records were examined for demographic, clinical, and laboratory data. *Results*: The coinfected group was younger, with 65% under 40 years, and presented significantly higher IL-6, PCT, and transaminase levels. Coexisting COPD and type 2 diabetes were independent predictors of coinfection. A higher SpO2 at diagnosis was positively associated with coinfection likelihood (OR = 5.37), while CT scores indicated less pulmonary involvement in coinfected patients. Non-fatal outcomes were more frequent in the coinfection group (95.7% sensitivity), and only one coinfected patient had a fatal outcome versus 17 in the SARS-CoV-2-only group. Low SpO2 and elevated IL-6 were significant predictors of mortality, with severe symptoms tripling fatality odds. *Conclusions*: Coinfection with extraPTB and SARS-CoV-2 is associated with younger age, heightened systemic inflammation, and longer hospital stays but does not significantly increase mortality risk compared to SARS-CoV-2 alone. These findings underscore the importance of monitoring systemic inflammatory markers and developing tailored management strategies to improve long-term care outcomes for coinfected patients, especially in resource-limited settings.

## 1. Introduction

The COVID-19 pandemic has significantly affected tuberculosis control efforts, leading to a sharp decline in case notifications due to disruptions in services. For the first time in over a decade, the mortality rate associated with tuberculosis has risen, as outlined in the World Health Organization Global Tuberculosis Report [1].

SARS-CoV-2, first identified in China in December 2019, was declared a pandemic by the World Health Organization on 11 March 2020 [2]. As of early 2024, over 774 million confirmed cases and more than 7 million deaths have been reported globally [3]. Preventive measures like masking and social distancing not only reduced COVID-19 transmission but also led to a notable decline in other respiratory infections, such as influenza and pneumococcal diseases [4]. The COVID-19 pandemic significantly disrupted tuberculosis (TB) control programs, causing a global decline in case reporting and limited access to care. This hindered the achievement of TB targets set by the United Nations [1]. While most TB cases result from reactivation of latent infections years after the initial infection, the pandemic’s immediate impact on TB rates was minimal [5].

It remains uncertain whether COVID-19 triggers the reactivation of latent TB or whether TB infection increases susceptibility to SARS-CoV-2. Some researchers hypothesize that SARS-CoV-2 may provoke an exaggerated inflammatory response, potentially activating latent or hidden infections. Additionally, immunosuppressive treatments for COVID-19 could unmask TB, as these medications are known to reactivate mycobacterial infections [6].

Roughly one-quarter of the global population is estimated to harbor a latent Mycobacterium tuberculosis infection, with 5–15% eventually progressing to active TB during their lifetime. The risk of reactivation varies depending on geographic and individual factors [7].

The ongoing SARS-CoV-2 pandemic continues to cause widespread illness and death, with COVID-19 primarily affecting the respiratory system but also potentially damaging other organs. Meanwhile, TB remains a prevalent disease, primarily affecting the lungs but occasionally manifesting in extrapulmonary forms, involving organs such as the meninges, lymph nodes, gastrointestinal tract, pleura, genitourinary system, and bones [8].

After the Mycobacterium tuberculosis (MTB) enters the body, the immune system launches a combination of innate and adaptive responses, which may clear the bacteria or allow the infection to persist, with or without T cell involvement. For MTB to establish an infection, it must first bypass the airway’s protective barriers and reach the lungs, where it infects alveolar macrophages, neutrophils, and dendritic cells. These infected cells then activate and recruit innate and adaptive lymphocytes to assist in containing the bacteria [9]. Natural killer (NK) cells play a crucial role by recognizing and destroying MTB-infected macrophages, enhancing IFN-γ production, and secreting cytokines that attract CD8+ T cells and NK T cells. This process facilitates the formation of granulomas, which consist of macrophages, neutrophils, and Langhans giant cells (formed from fused macrophages) and are surrounded by lymphocytes and a fibrotic layer [10].

Approximately 25% of pulmonary TB cases progress to extrapulmonary TB, which can affect any organ. Diagnosis often requires invasive procedures and imaging studies due to the wide range of symptoms, leading to delays from symptom onset to microbiological confirmation [11]. There is limited research on the co-occurrence of extrapulmonary TB and COVID-19, highlighting a significant gap in our understanding [12]. Extrapulmonary TB (extraPTB) does not typically present with the common symptoms observed in pulmonary TB (PTB) patients, such as cough, weight loss, hemoptysis, and night sweats. Instead, symptoms vary based on the affected organ system and may include joint pain, abdominal discomfort, headaches, diarrhea, or lymphadenopathy. ExtraPTB frequently affects the lymphatic system, particularly in individuals with compromised immune systems. Moreover, patients with extraPTB may have normal chest radiographs, which can further complicate diagnosis [13]. Recent research has explored the relationship between SARS-CoV-2 and host protein interactions, revealing that MTB shares many of the same host protein interaction partners as SARS-CoV-2. This finding is particularly relevant given that both diseases primarily affect the lungs, increasing the risk of compounded respiratory complications [14].

After having previously analyzed the impact of SARS-CoV-2 infection on patients with pulmonary TB infection [15], this study comes as a continuation of our team’s work to assess the consequences of SARS-CoV-2 infection in patients with extraTB. This work uniquely contributes to the literature by focusing on the clinical dynamics of SARS-CoV-2 coinfection in patients with extraPTB, which remains under-researched compared to the more commonly studied pulmonary TB.

## 2. Materials and Methods

The present retrospective, cross-sectional, randomized study involved 123 adult patients aged 19 to 91 years, hospitalized at the Victor Babeș Hospital of Infectious Diseases and Pneumoftiziology from Timișoara between March 2020 and March 2022, during the COVID-19 pandemic.

### 2.1. Study Design and Objectives

This study aimed to compare two groups of patients: one group with extrapulmonary tuberculosis (extraPTB) and SARS-CoV-2 coinfection (n = 23) and a control group of age-matched patients diagnosed only with moderate or severe SARS-CoV-2 infection (n = 100). The primary objective was to evaluate the impact of SARS-CoV-2 on patients already burdened by tuberculosis.

### 2.2. Inclusion Criteria

Patients in the following situations were included in this study:

❖ Diagnosed with extrapulmonary TB within one month of SARS-CoV-2 infection confirmation using solid or liquid cultures (Gene-Xpert) at the TB ambulatory service in Timișoara [16].
❖ Diagnosed with moderate or severe forms of SARS-CoV-2 at hospital admission, confirmed by RT-PCR from nasopharyngeal swabs in an accredited laboratory. Moderate infection was characterized by signs of lower respiratory disease or imaging findings with SpO2 ≥ 94% on room air. Severe infection was defined by SpO2 < 94% on room air, a respiratory rate > 30 breaths/min, or >50% lung infiltrates [17].
❖ Normal renal function (normal GFR, creatinine, and urea levels) [18].
❖ Prior BCG vaccination [19].

### 2.3. Exclusion Criteria

Patients presenting the following were excluded:

❖ Overweight (BMI = 25–30 kg/m^2^) or obesity (BMI ≥ 30 kg/m^2^) [20].
❖ Pre-existing severe or uncontrolled arterial hypertension [21].
❖ Pre-existing advanced chronic heart failure [22].
❖ Any type of neoplasia [23,24].
❖ Chronic pulmonary, hepatic, renal, or digestive conditions and HIV infection.

Exclusions for obesity and chronic conditions were applied to reduce confounding effects on inflammatory markers, which are independently elevated in these conditions, as well as on COVID-19 outcomes. While these exclusions might limit the generalizability to populations with metabolic or chronic comorbidities, they allow for a clearer analysis of coinfection dynamics between SARS-CoV-2 and extraPTB [25].

Ethical Considerations: This study received approval from the Ethics Council for Scientific Research at the Victor Babeș University of Medicine and Pharmacy Timisoara, adhering to the principles of the Helsinki Declaration (04/19 January 2021). Informed consent was obtained from all participants after explaining the nature of the data analysis.

Data Collection: Data were collected from personal medical files, including anamnesis, clinical parameters, and biological and imaging investigations.

Medical History: BCG vaccination status, comorbidities such as COPD and type 2 diabetes, and smoking status (never smoked, smoker) were documented. Employment status was categorized into unemployed, employed, and retired. No subject included in this study had ever been diagnosed and treated for tuberculosis.

Measurements: BMI was calculated using the formula weight (kg)/height^2^ (m^2^) [26]. SpO2 (%) values at the time of SARS-CoV-2 diagnosis and the lowest SpO2 (%) values during hospitalization were recorded using calibrated pulse oximeters [27]. Peripheral systolic and diastolic blood pressures (SBP, DBP, mmHg) were noted at diagnosis.

Symptoms: The SARS-CoV-2 symptoms recorded included fever, coughing, dyspnea, fatigue, abdominal pain, chest pain, myalgia, vomiting/nausea, diarrhea, headache, and olfactory/taste disorders. The symptoms were classified as more severe if accompanied by tachypnea (respiratory rate ≥ 30 breaths/min) [28].

Blood Markers: C-reactive protein (CRP, mg/L); procalcitonin (PCT); aspartate aminotransferase (AST, U/L); alanine aminotransferase (ALT, U/L); lactate dehydrogenase (LDH, U/L); interleukin-6 (IL-6, pg/mL); D-dimer levels (mg/L); and neutrophil, lymphocyte, and platelet counts (/µL) were recorded. The ratios calculated included the neutrophil-to-lymphocyte ratio (NLR), platelet-to-lymphocyte ratio (PLR), and systemic immuno-inflammatory index (SII) [29,30,31].

Imaging: An experienced radiologist reviewed all CT scans at SARS-CoV-2 infection confirmation. CT scans were assessed for lesions such as ground-glass opacifications, consolidations, crazy paving pattern, linear opacities, air bronchogram sign, tree in bud, and cavitary lesions [32,33]. The extent of lung involvement was scored from 0 to 5 for each lobe, with a total possible score ranging from 0 to 25, providing a comprehensive view of lung disease impact [3,34,35].

Outcome: The number of hospitalization days and patient outcomes (resolved-PCR converted or deceased) were recorded for each case.

### 2.4. Data Analysis

Data collection was carried out using Microsoft Excel, while statistical analyses were performed with MedCalc Statistical Software version 20.111 (MedCalc Software Ltd., Ostend, Belgium). The primary aim of the analysis was to examine the impact of SARS-CoV-2 infection on patients with and without PTB coinfection. Clinical, imaging, and biological parameters were compared between the groups. Statistical significance was defined as *p*-values below 0.05. To assess the normality of the data distribution, the Shapiro–Wilk test was utilized. Based on the results of this test, appropriate statistical methods were chosen: medians and the Mann–Whitney test were used for non-normally distributed variables. AUC–ROC analysis was employed to evaluate the significance of various parameters in distinguishing between the presence and absence of coinfection, with cut-off values determined to highlight the importance of specific parameters in these medical conditions. Logistic regression models were used to identify independent predictors of coinfection and fatal outcomes. The associations between binary datasets were evaluated using Fisher’s exact test. Variance inflation factors (VIFs) were calculated for all predictors in the logistic regression model. All VIFs were below 5, indicating no significant multicollinearity. Missing data were minimal (<5%) and were handled using complete case analysis, given the limited sample size. This approach ensures the integrity of the dataset while avoiding bias introduced by imputation methods.

## 3. Results

This study included 123 patients, divided into two study groups comprising 23 patients with extraPTB and SARS-CoV-2 coinfection and 100 patients with SARS-CoV-2 infection alone.

The patients were distributed relatively homogenous regarding sex in both groups. Regarding age, the younger subjects were in the majority in the coinfection group, 65% under 40 years old, while in the SARS-CoV-2 group, the subjects aged 40 years and older comprised 89% of cases (Table 1). The mean age in the coinfected group was 40.1 years (SD = 18.6), while the median age in the SARS-CoV-2 group was 56.5 years.

**Figure 1:**
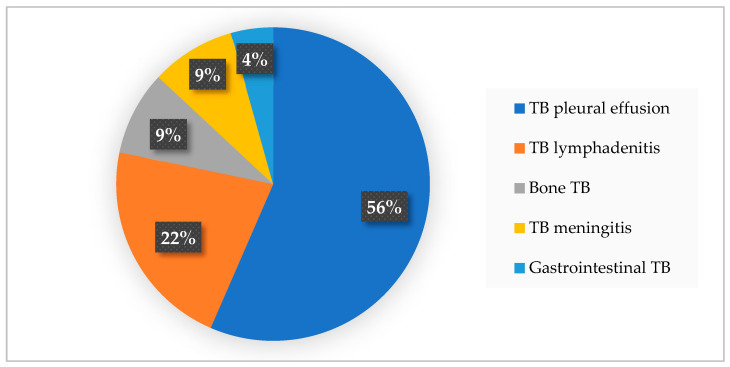
Types of extraPTB included in this study.

The subjects in the extraPTB group were diagnosed with different types of TB infections: a total of thirteen subjects (56%) presented TB pleural effusion, five subjects (22%) presented TB lymphadenitis, two subjects (9%) presented TB meningitis, two subjects (9%) presented bone TB, and one subject (4%) presented gastrointestinal TB (see Figure 1). Only four of all cases of extraTB had previously undergone immunosuppressive therapies: one with pleural effusion and two with lymphadenitis.

**Figure 2:**
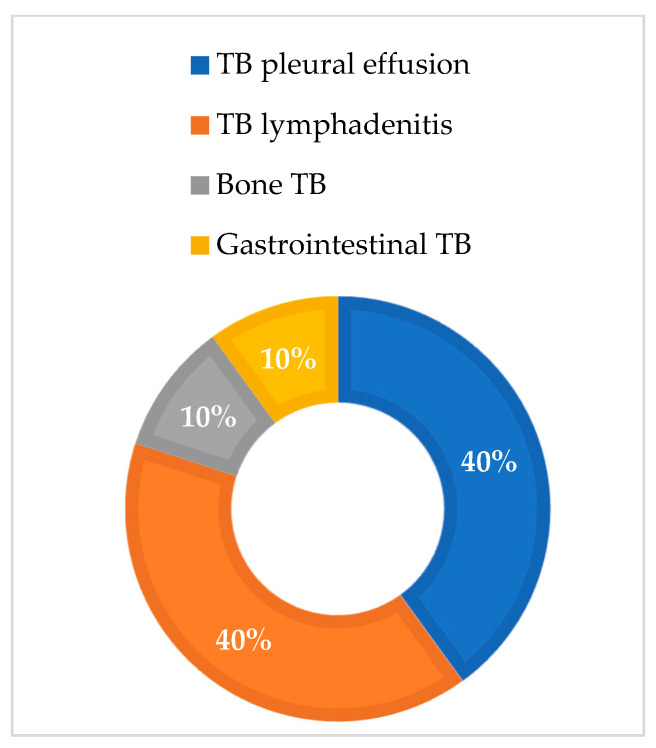
ExtraPTB cases in females.

**Figure 3:**
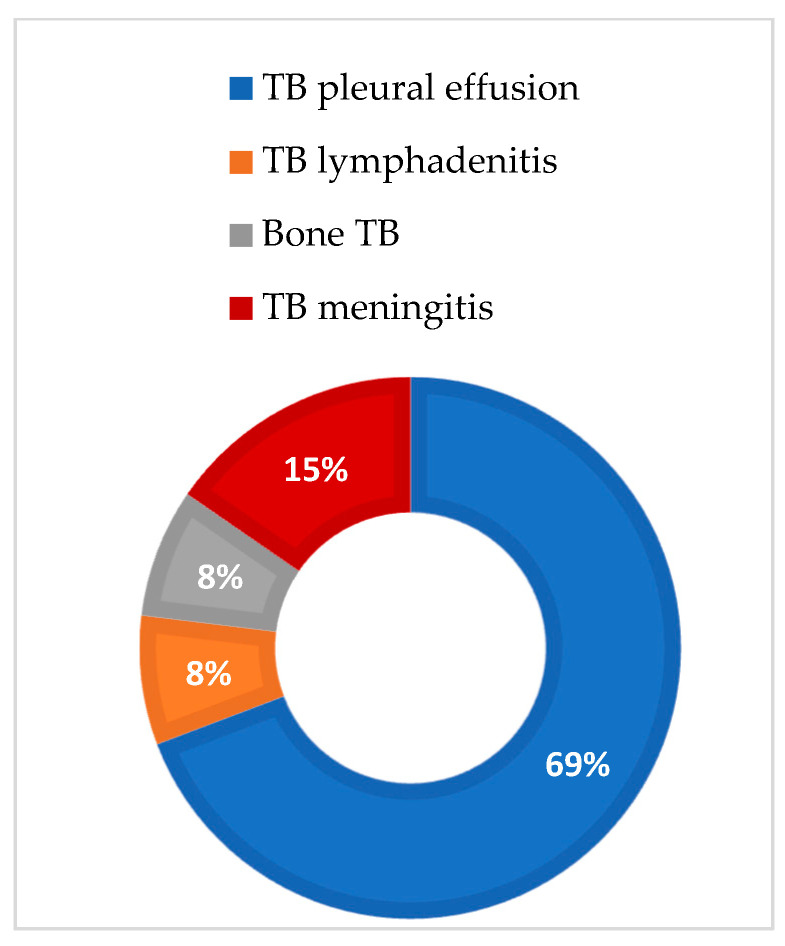
ExtraPTB cases in males.

When analyzing sex-based differences regarding the proportion of extraPTB cases, in women, TB pleural effusion and TB lymphadenitis were the most predominant types of TB infections, with equal proportions of 40% (Figure 2), while in men, 69% of cases were of TB pleural effusion, followed by 15% TB meningitis (Figure 3).

A comparison between the extraPTB subtypes was performed in order to analyze the differences and understand subgroup dynamics: pleural effusion TB (thirteen subjects) vs. TB lymphadenitis (five subjects).

Patients with pleural TB were significantly older than those with lymphadenitis TB (*p* = 0.03, Mann–Whitney U test). There was no statistically significant difference in the proportion of smokers between patients with pleural TB and those with lymphadenitis TB (*p* = 0.72, Fisher’s exact test).

Pleural TB patients had significantly lower SpO2 values at admission compared to lymphadenitis TB patients (*p* = 0.03). This suggests greater respiratory compromise in pleural TB cases. The median CRP levels (*p* = 0.006) and median IL-6 levels (*p* = 0.04) were significantly higher in patients with pleural TB compared to lymphadenitis TB. There was a statistically significant difference in the length of hospitalization between the two groups, with pleural TB patients having longer stays (*p* = 0.002). This may reflect the higher severity or more complex management of pleural TB cases. The Mann–Whitney U test was used for these continuous variables.

No significant difference was observed in outcomes (resolved or deceased) between pleural TB and lymphadenitis TB patients (*p* = 0.65, Fisher’s exact test). This indicates that the clinical outcome was not associated with the type of extrapulmonary TB in this dataset.

**Figure 4:**
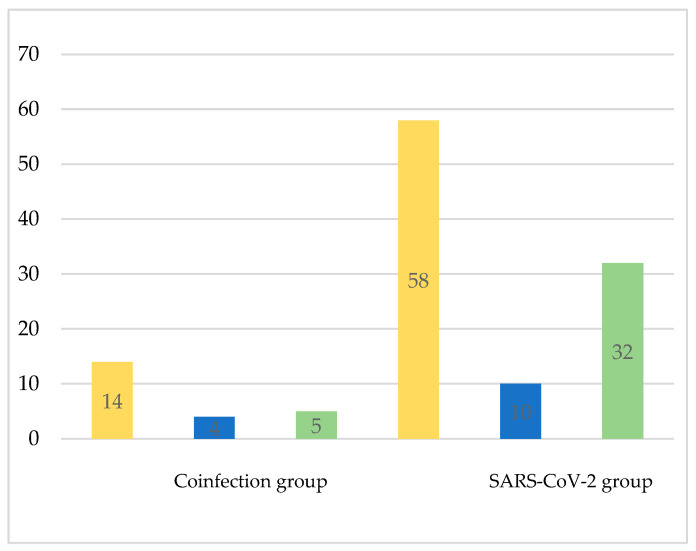
Distribution of cases according to the social status (yellow—no. of employed subjects; blue—no. of unemployed subjects; green—no. of retired subjects).

Regarding social status, 60.9% of the subjects in the coinfection group vs. 58% of the SARS-CoV-2 group were employed, and, respectively, 17.4% vs. 10% were unemployed, and 21.7% vs. 32% were retired (Figure 4).

**Figure 5:**
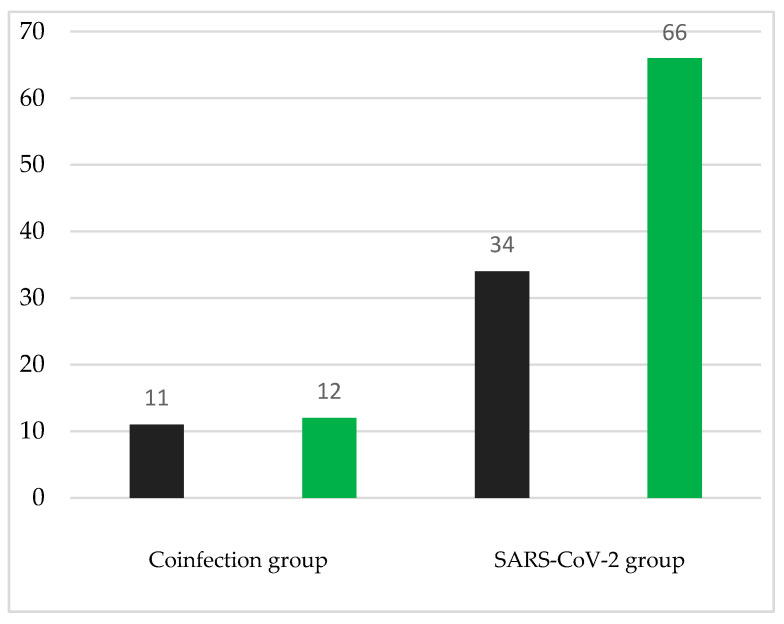
Distribution of cases according to smoking (black—no. of smokers; green—no. of non-smokers).

Regarding smoking, the coinfection group consisted of similar proportions: a total of 47.8% of subjects having declared they were regular smokers and 52.2% having declared that they never had been smokers. In the SARS-CoV-2 group, approximately one-third of the subjects (34%) declared that they were smokers (Figure 5).

The comparison revealed significant differences between the two groups in most of the analyzed parameters. The SARS-CoV-2 group revealed significantly lower SpO2 both at diagnosis and with regard to the lowest value registered. Moreover, the chest CT involvement score revealed a significantly higher median score for the subjects pertaining to the SARS-CoV-2 infection group. In contrast, the coinfection group displayed higher IL-6, transaminase, and D-dimer median values and also higher NLR, PLR, and SII median values. No differences were detected with regard to blood pressure levels (Table 2).

A multiple parameter logistic regression was employed with the dependent variable being the presence/absence of extraPTB–SARS-CoV-2 coinfection and the independent variables being age, sex, BMI, employment status, smoking status, associating COPD, and type 2 DM. Age, employment status, associating COPD, and type 2 DM emerged as significant predictors of extraPTB–SARS-CoV-2 coinfection. As age increased, the likelihood of having extraPTB and COVID-19 coinfection slightly decreased (13%). The median age difference in the two study groups should be taken into consideration as per this result. Being employed was associated with 75% higher odds of having the coinfection. Individuals with COPD had 6.5 times higher odds of having the coinfection compared to those without COPD. Subjects with type 2 DM had over four times higher odds of having the coinfection compared to those without diabetes. We must mention, however, the large distribution of the data in the cases of coinfected subjects who associated COPD (95% CI 1.12–37.71) or type 2 DM (95% CI 1.1–39.66). We attribute these results to the small sample sizes and suggest this should be considered as a limit in interpreting the ORs. Sex, BMI, and the status of smoking were not retained by the model (see Table 3). In the coinfection group, four subjects (17.4%) associated COPD, and three (13%) associated type 2 DM. Only one subject associated both conditions. In the SARS-CoV-2 group, an equal proportion of 19% presented COPD and diabetes.

A logistic regression was also employed to include paraclinical parameters as independent variables. The presence/absence of extraPTB–SARS-CoV-2 coinfection was still considered the dependent variable, while the independent variables were represented by the SpO2 at diagnosis, lowest SpO2, SBP, DBP, CRP, LDH, PCT, IL-6, AST, ALT, D-dimer levels, neutrophil count, lymphocyte count, thrombocyte count, NLR, PLR, and SII. The SpO2 at diagnosis, the PCT, and the CT involvement score were deemed significant independent predictors for the presence of coinfection. Due to the fact that the coinfection group presented significantly higher SpO2 levels at diagnosis, the model revealed that a higher SpO2 at diagnosis is positively associated with the likelihood of having the coinfection (OR = 5.37). Moreover, also attesting to less pulmonary involvement in patients with coinfection, with each unit increase in the CT involvement score, the odds of having the coinfection decreased by 51%. This suggests that higher CT involvement scores are associated with higher odds of having the COVID-19 infection alone. A positive PCT is associated with an increased likelihood of having the coinfection, with patients presenting a positive PCT having a 6.5 times higher likelihood of being coinfected. The rest of the independent variables were not retained by the model (Table 4).

Further on, AUC–ROC analyses were employed in order to determine the significance of multiple parameters in discriminating between the presence and absence of extraPTB coinfection (Table 5).

**Figure 6:**
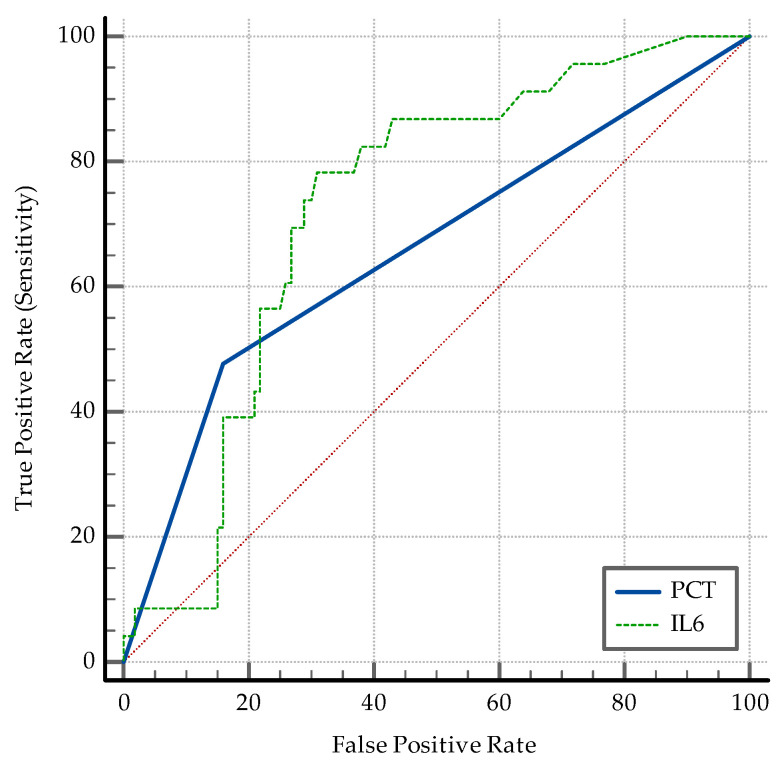
ROC curve for the significant inflammatory markers (PCT and IL6).

**Figure 7:**
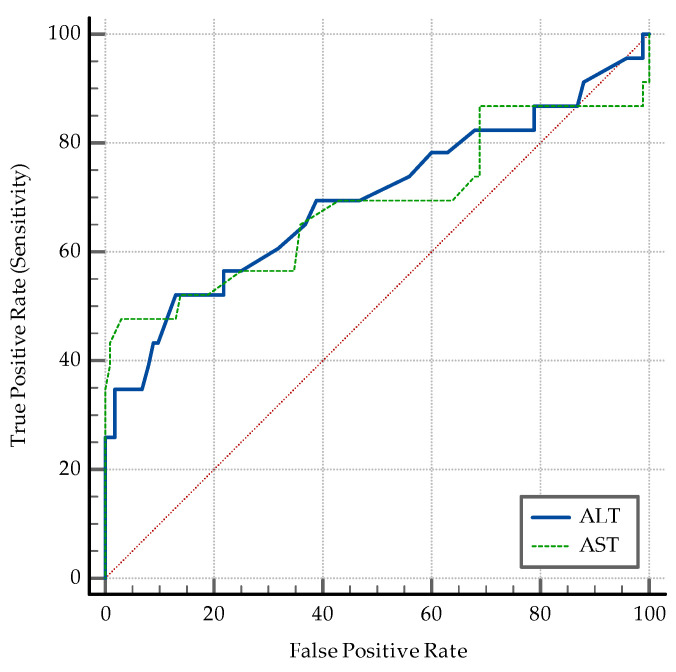
ROC curve for transaminase levels.

**Figure 8:**
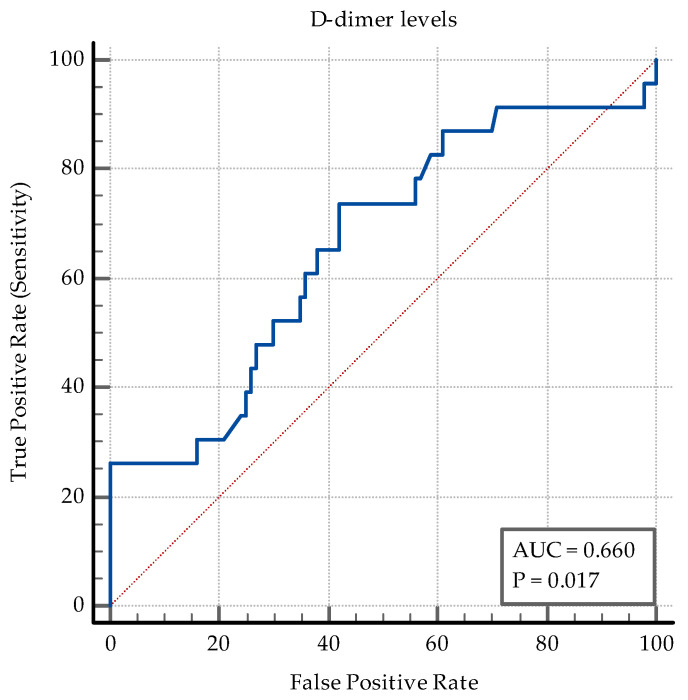
ROC curve for D-dimer levels.

**Figure 9:**
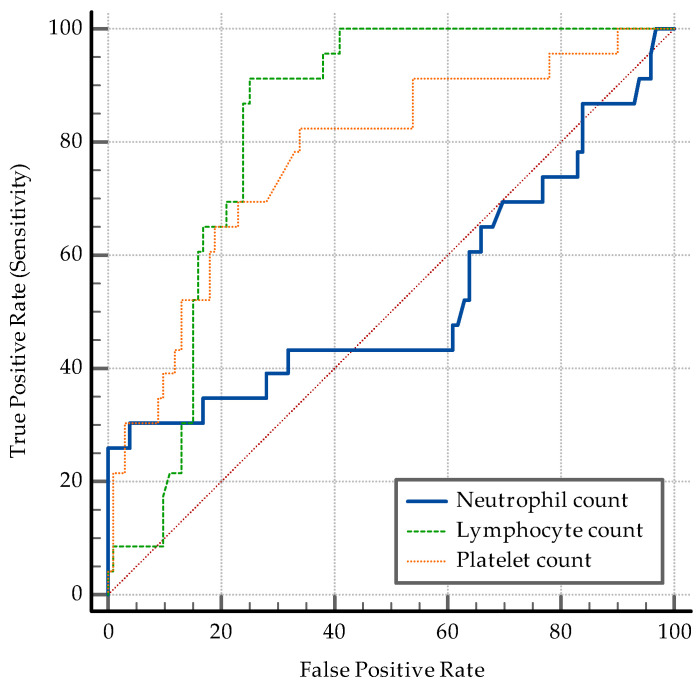
ROC curve for blood cell count.

**Figure 10:**
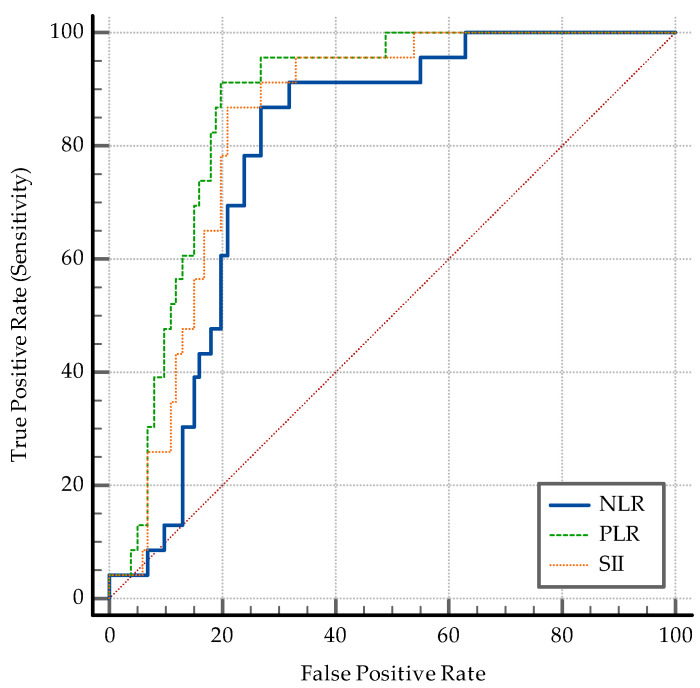
ROC curve for NLR, PLR, and SII.

**Figure 11:**
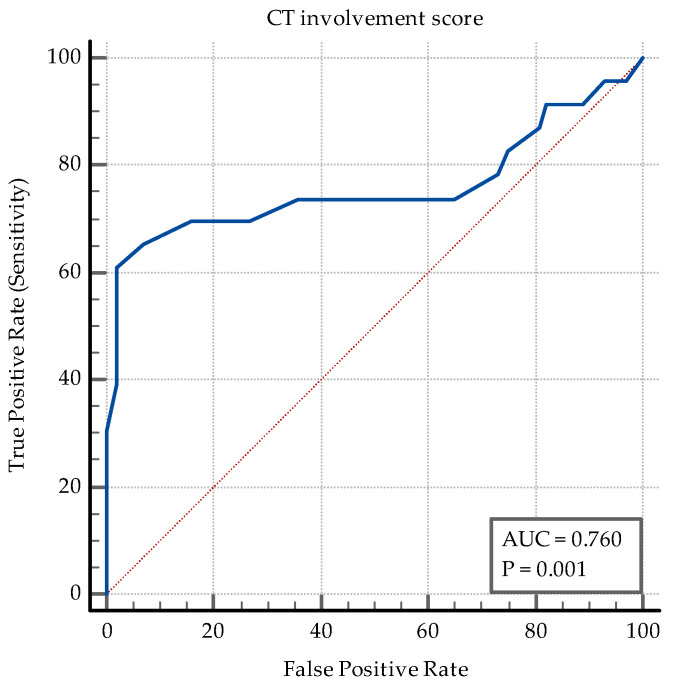
ROC curve for the CT involvement score.

The AUC–ROC analysis showed that younger age and being employed are significant discriminators. An SpO2 > 95% detects coinfection with a specificity of 77%. A positive PCT and an IL-6 > 3.6 pg/mL are both significant (Figure 6). While PCT has a high specificity of 84% and a relatively low sensibility (47.83%) in discriminating between coinfection and SARS-CoV-2 infection alone, IL-6 has an Se of 78.26% and an Sp of 69%. The transaminase levels also have significant value as discriminators: ALT having an Sp of 97% while AST having an Sp of 87% (Figure 7). High D-dimer levels also displayed significance: a level > 1.1 mg/L displaying an Se of 73.91% but a lower Sp (Figure 8). As per the blood cell count and the ratios calculated as inflammation markers, all are significant detectors of coinfection. High neutrophil and platelet counts and a lower lymphocyte count are associated with coinfection (Figure 9). High NLR, PLR, and SII are also associated with coinfection (Figure 10). As per the CT involvement score, as the extraTB group has displayed a lower pulmonary involvement, a CT score < 6 was associated with an Sp of 98% in detecting coinfection (Figure 11).

Figure 6 demonstrates the diagnostic utility of inflammatory markers (PCT and IL-6) in distinguishing coinfection, with both markers showing moderate to high sensitivity.

Increased ALT and AST levels highlight the role of liver dysfunction in patients with coinfection, with both transaminases displaying high specificities (Figure 7).

Elevated D-dimer levels reflect enhanced coagulation activity in coinfected patients, with these levels displaying a moderately-high sensitivity (Figure 8).

Neutrophil and platelet counts are higher in coinfection, indicating enhanced systemic inflammation, while lymphocyte counts are reduced (Figure 9).

Higher NLR, PLR, and SII values demonstrate significant inflammatory and immune responses in coinfected patients (Figure 10).

Lower CT involvement scores in coinfection suggest reduced pulmonary involvement compared to SARS-CoV-2 infection alone (Figure 11).

Fisher’s exact test determined that, out of the binary data analyzed, regarding the connection between coinfection and the following parameters, the presence of COPD, type 2 DM, and a positive PCT have been significantly associated with the increased likelihood of coinfection (Table 6).

**Figure 12:**
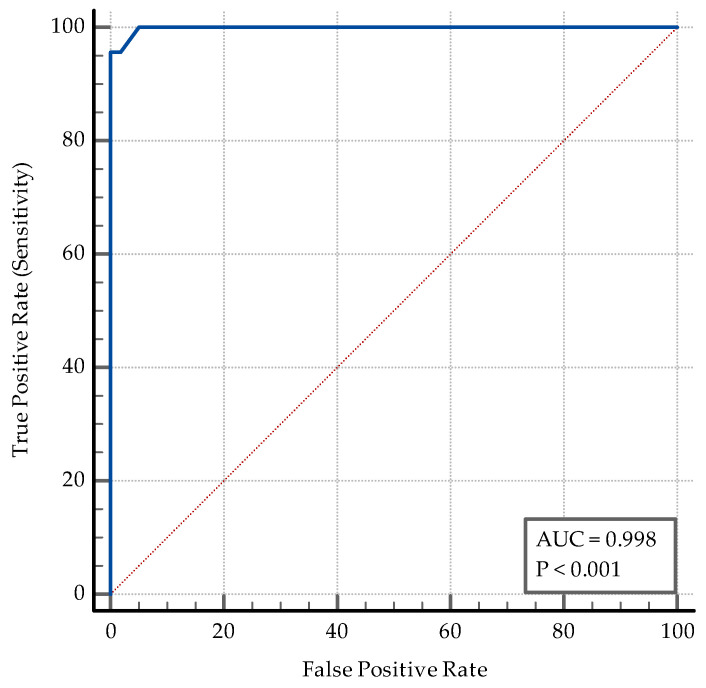
ROC curve for the hospitalization period.

The median hospitalization period was 30 days (limits: 15–60 days) for the subjects with coinfection and only 8 days (limits: 4–17 days) for the subjects with SARS-CoV-2 infection alone. A hospitalization period larger than 17 days can predict the coinfection status with a sensibility of 95.65% (95% CI = 78.1–99.9) and a specificity of 100% (95% CI = 96.4–100), AUC = 0.99, *p* < 0.0001, in our cohort (Figure 12).

**Figure 13:**
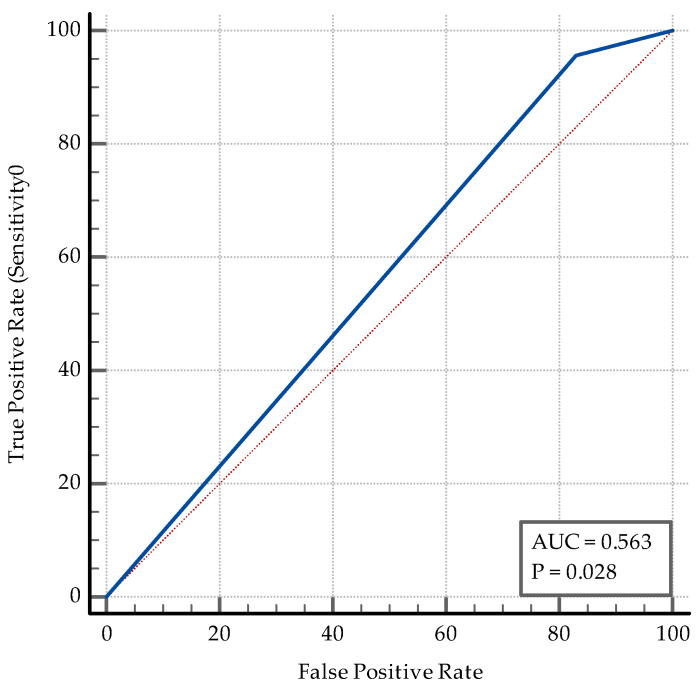
ROC curve for outcomes.

While 17 subjects in the SARS-CoV-2 group had fatal outcomes, only one subject in the coinfection group had the same outcome. The AUC–ROC analysis showed that a non-fatal outcome is a reliable predictor of coinfection, with a very high sensitivity (Se = 95.65%, 95% CI = 78.1–99.9) but a low specificity (Sp = 17%, 95% CI = 78.1–99.9), AUC = 0.56, *p* = 0.02 (Figure 13).

A logistic regression was employed with the status of either coinfection or SARS-CoV-2 infection alone, sex, smoking status, COPD and type 2 DM association, and the gravity of symptoms as independent variables. The outcome represented the dependent variable. The results showed the severity of symptoms increases the probability of a fatal outcome by three-fold (OR = 3.21, *p* < 0.0001). The status of coinfection is not a predictor of fatality. The rest of the parameters were not retained by the model.

Another logistic regression was employed to evaluate the effects of several non-binary markers (independent variables: age, BMI, SpO2 at diagnosis and the lowest SpO2, CT involvement score, CRP, PCT, IL-6, LDH, ALT, AST, Ne count, Ly count, Tr count, D-dimer levels, NLR, PLR, SII, and hospitalization period) on outcome (dependent variable). The result showed that the lowest value of SpO2 registered and IL-6 are significant independent predictors of a fatal outcome. The rest of the variables were not retained by the model (Table 7).

## 4. Discussions

This study builds on our prior research regarding the effects of SARS-CoV-2 on patients with pulmonary TB [15] by shifting the focus to those with extrapulmonary tuberculosis (extraPTB). It aims to examine how COVID-19 affects extraPTB patients compared to those infected only with SARS-CoV-2, contributing to the broader understanding of the interaction between TB and COVID-19, with an emphasis on these less-explored forms of TB. To our knowledge, a study focusing on precisely that has not been published before. Two groups were compared: one with extraPTB and SARS-CoV-2 coinfection and a group with only SARS-CoV-2 infection. The discussion will focus on the key findings and their implications in understanding the interaction between these two infectious diseases.

### 4.1. Key Findings

The results demonstrated significant differences between the coinfected group and the group with SARS-CoV-2 infection alone, both in terms of clinical and laboratory parameters. Coinfected patients exhibited higher levels of inflammatory markers, such as IL-6, CRP, and systemic inflammatory indices (NLR, PLR, SII), as well as D-dimer and transaminase levels. The coinfected patients also displayed a significantly lower lymphocyte count. A positive PCT was associated with a 6.5 times increase in the likelihood of having coinfection. Although these results suggest an amplified inflammatory response in coinfected patients, the symptomatology and outcomes were contrary. Interestingly, the SARS-CoV-2 group presented with more severe hypoxemia (lower SpO2 levels at diagnosis and during hospitalization) and more extensive lung involvement, as evidenced by higher chest CT scores. An explanation could be that patients with extraPTB typically have less pulmonary involvement than those with pulmonary TB. This finding is consistent with studies that have identified less severe lung disease in patients with extrapulmonary forms of TB, particularly when coinfected with other respiratory pathogens [36].

These findings indicate that, while patients with extraPTB may present with less severe respiratory symptoms, they may still be at risk of severe systemic inflammation, which requires careful monitoring. Elevated PCT and IL-6 levels, in particular, have been previously identified as markers of severe COVID-19 outcomes, and their role in the coinfected population needs further exploration [37]. It has been shown that some of the markers of COVID-19 deterioration and the development of acute respiratory distress syndrome include elevated LDH, CRP, IL-6, D-dimer levels, lymphocytes, platelets, renal function, and also high-sensitive troponin. Moreover, critically ill patients often exhibit significant lymphopenia and neutrophilia, leading to an increased NLR [37].

One explanation for the less severe respiratory lesions in patients with coinfection could be the activity of anti-inflammatory regulatory T cells (Treg) in extraPTB. Treg cells tend to increase, especially in cases of active TB. These cells likely expand in response to heightened inflammation rather than causing the disease. Treg cells, a subset of CD4+ T lymphocytes (1–5% of circulating CD4+ cells), prevent autoimmunity and maintain immune balance [38,39,40]. Treg cells may help limit excessive immune responses, which could prevent tissue damage, but this suppression also allows for the pathogen to persist. Studies show that patients with latent TB infection may have higher Treg levels, which could protect against progression to active TB, while those with active TB show an increase in Treg cells over time [41]. Depleting Treg cells enhances immune responses to pathogens like Helicobacter pylori, HIV, hepatitis C virus, MTB [42], and also SARS-CoV-2 [43].

For instance, Treg depletion experiments in mouse models of TB have demonstrated increased production of pro-inflammatory cytokines, such as TNF-α and IFN-γ, resulting in heightened bacterial clearance but at the cost of increased tissue pathology [41]. Similarly, in SARS-CoV-2, lower Treg activity has been linked to excessive cytokine storms, contributing to respiratory failure in severe COVID-19 cases. This reduction may affect patients by weakening inflammatory control, disrupting the Treg/Th17 balance, and increasing the risk of respiratory failure [43,44]. Interestingly, the Treg/Th17 balance is a key factor in immune regulation during coinfections. Th17 cells promote inflammation, which is crucial for pathogen clearance but can also lead to tissue damage if unchecked. An imbalance skewed toward Th17 dominance, as observed in some severe COVID-19 cases, has been associated with worse outcomes [44,45]. In contrast, Treg predominance, as is often observed in extraPTB, might suppress this excessive inflammatory response, potentially explaining the less severe respiratory lesions in coinfected patients. However, it remains unclear whether reduced Tregs lead to poor outcomes or if higher Treg levels are beneficial. Many researchers agree that maintaining a balanced Treg count is crucial in mitigating the adverse effects of severe SARS-CoV-2 infection [44,45,46].

Further, the role of Tregs in mediating systemic versus localized immune responses is increasingly recognized. In the case of extraPTB, Tregs may preferentially act on systemic inflammation, sparing the lungs from excessive immune-mediated damage while controlling the extrapulmonary spread of Mycobacterium tuberculosis. This systemic modulation may offer partial protection against the widespread pulmonary inflammation characteristic of severe SARS-CoV-2 infection [41,44,45].

### 4.2. Demographic Factors, Age, and Comorbidities

The key factors traditionally linked to the development of extraPTB are being at either end of the age spectrum, female sex, migration from countries with high TB prevalence, and having a compromised immune system [47]. Global epidemiology studies place extraPTB at 20–30% of cases of TB infection, depending on geographical regions [48].

The demographic characteristics of our study population showed a slight predominance of males in both groups, which is consistent with the global epidemiology of TB and COVID-19 but not of extraPTB [49,50]. Still, epidemiological reports regarding sex-based differences in extraPTB are inconclusive. A large study in Mali, which included 1012 subjects with confirmed extraPTB, reported a male-to-female ratio of 1.59:1 [51]. This study also found differences regarding the affected organ: males are more likely to present pleural TB (OR = 1.51), and women are more likely to present lymph node (OR = 0.57) and abdominal TB (OR = 0.59) [51]. In contrast, a large Korean study places females aged 40 to 60 years at a greater risk of presenting with extraPTB than men [52]. A study from Madrid also showed that women are more likely than men to develop extraPTB. They also associated female sex with an increased likelihood of presenting lymphatic and pleural TB, while males were more likely to present genitourinary and abdominal TB [11]. Furthermore, Pang et al. examined the epidemiology of extrapulmonary TB among inpatients in China, concluding that the most common forms of extrapulmonary TB were osteoarticular and pleural. Most of these diagnoses were based on clinical symptoms, indicating a high risk of diagnostic delays and misdiagnoses in extrapulmonary TB cases [53]. In our study, the subjects presented different types of extraTB infections: a total of thirteen subjects (56%) presented TB pleural effusion, five subjects (22%) presented TB lymphadenitis, two subjects (9%) presented TB meningitis, two subjects (9%) presented bone TB, and one subject (4%) presented gastrointestinal TB. Only four of all cases of extraTB had previously undergone immunosuppressive therapies: one with pleural effusion and two with lymphadenitis. Only four of all cases of extraTB had previously undergone immunosuppressive therapies, one with pleural effusion and two with lymphadenitis. When analyzing sex-based differences regarding the proportion of extraPTB cases, in women, TB pleural effusion and TB lymphadenitis were the most predominant types of TB infections, with equal proportions of 40%, while in men, 69% of cases were of TB pleural effusion, followed by 15% TB meningitis.

The logistic regression analysis revealed that younger age, employment status, and comorbidities such as COPD and type 2 diabetes were significant predictors of extraPTB–SARS-CoV-2 coinfection.

The observation that the extraPTB and SARS-CoV-2 coinfection group in this study was younger than the SARS-CoV-2-only group raises intriguing questions about the role of age in immune responses and clinical outcomes. The negative correlation between age and coinfection may reflect the fact that younger individuals are more likely to develop extraPTB due to their more robust immune responses, while older adults are more prone to pulmonary forms of TB [54]. Young adults, especially those with strong cell-mediated immunity, are more likely to contain the infection in specific sites outside the lungs, leading to localized extrapulmonary manifestations, such as lymph node, pleural, or bone TB. Elders, on the other hand, may experience more disseminated disease, but their overall risk for extraPTB tends to be lower compared to younger adults [55]. Hence, while pulmonary TB is more common in older populations due to the reactivation of latent infections, extraPTB tends to be more prevalent in younger individuals, especially in areas with a high burden of TB [10]. When considering the coinfection with SARS-CoV-2, younger individuals also seem to exhibit better pulmonary outcomes despite the heightened systemic inflammation associated with extraPTB. The improved respiratory outcomes in younger populations with SARS-CoV-2 infection can be attributed to stronger adaptive immune responses. Studies show that younger individuals have higher baseline T cell activity, including cytotoxic CD8+ T cells and helper CD4+ T cells, which play a crucial role in clearing viral infections [56]. This enhanced immune function allows for faster resolution of viral replication and better control of inflammation, limiting pulmonary damage despite the systemic inflammatory milieu associated with extraPTB. Moreover, younger individuals generally have a more robust production of type I and III interferons, which are critical in the early antiviral response [57]. These interferons not only limit viral spread but also modulate the inflammatory response, preventing excessive cytokine release that can lead to acute respiratory distress syndrome in severe COVID-19 cases. This balance between effective immune activation and controlled inflammation likely contributes to the less severe pulmonary involvement observed in the younger coinfected group.

This may be the explanation for the difference in median age between the two groups in our study, with the coinfection group including significantly younger subjects than the SARS-CoV-2 group. Being employed was also associated with a higher probability of having extraPTB, but we associate this result with the younger median age of the group.

This study suggests an association between COPD and type 2 diabetes mellitus and extraPTB coinfection. Individuals with COPD presented 6.5 times higher odds of having the coinfection, while those with type 2 DM had four times higher odds of having the coinfection. Although several previous studies have highlighted the role of these comorbidities in increasing the risk of tuberculous infection and in exacerbating the clinical course of COVID-19 [58,59,60,61], those studies have focused on either PTB or on tuberculous infection of either etiology combined. Data are scarce on the implications of these pathologies in extraPTB and SARS-CoV-2 coinfection, and our coinfection group is too small to draw definitive conclusions.

It is clear that COPD is primarily associated with pulmonary complications, leading to worse outcomes in PTB patients. Studies have shown that COPD patients are three times more likely to develop active TB, and when coinfected with SARS-CoV-2, the mortality and hospitalization rates increase significantly. The lung damage caused by COPD makes it easier for Mycobacterium tuberculosis to bypass immune defenses, leading to the development of active TB [58]. TB often leads to post-tubercular airway disease or TB-associated COPD, with COPD being a common comorbidity, second only to diabetes. Smoking accelerates COPD progression, increases TB risk by 3–5 fold, and exacerbates lung damage through inflammatory and proteolytic pathways. Patients with a TB history develop COPD earlier, face more hospitalizations, and have a shorter life expectancy [62]. Moreover, the immune dysregulation in COPD patients, exacerbated by smoking and environmental exposures, often leads to poorer control of both bacterial and viral infections. When these patients develop COVID-19, they are more likely to experience severe respiratory complications [59].

On the other hand, type 2 DM is another key comorbidity associated with adverse outcomes in both TB and COVID-19. Diabetic patients often exhibit impaired immune responses, making them vulnerable to both TB reactivation and severe manifestations of SARS-CoV-2 infection. In PTB and SARS-CoV-2 coinfections, DM exacerbates the clinical course, often leading to prolonged hospitalization and an increased risk for complications, including multi-organ failure and poor prognosis. Studies emphasize the importance of tight glycemic control during hospitalization to mitigate these risks [60,61].

These comorbidities may contribute to the complexity of managing extraPTB–SARS-CoV-2 coinfection. We highlight the need for targeted treatment protocols and further research to develop comprehensive management guidelines for these high-risk populations.

### 4.3. Hospitalization and Outcomes

This study also found that coinfected patients had significantly longer hospitalization periods compared to those with SARS-CoV-2 infection alone. This aligns with previous studies, which have reported longer recovery times in TB patients coinfected with COVID-19, likely due to the dual burden of managing both infections simultaneously. A recent meta-analysis reported that individuals with TB–COVID-19 coinfection are at heightened risk of hospitalization and protracted recovery periods [55]. Moreover, in patients with concurrent TB and COVID-19 infections, increased mortality rates and extended recovery times have been reported [63].

However, it is notable that mortality was significantly higher in the SARS-CoV-2-alone group compared to the coinfection group in our study. Coinfection was not a significant predictor of fatality, but the lowest value of SpO2 registered and IL-6 levels were. The severity of symptoms increases the probability of a fatal outcome by three-fold. This finding could be explained by the younger age and relatively better respiratory function in the extraPTB cohort, despite the systemic inflammation. It may also indicate that, while extraPTB exacerbates the inflammatory response, it does not necessarily lead to worse pulmonary outcomes compared to COVID-19 infection alone [64].

### 4.4. Limitations and Future Directions

While this study offers important insights into the interplay between extraPTB and SARS-CoV-2, certain limitations must be acknowledged. Firstly, the relatively small sample size of the coinfected group may limit the generalizability of the findings. Our pre-statistical estimations, however, showed that the cohorts were sufficiently large to provide statistical significance. To this point, we mention the large distribution of data in the cases of coinfected subjects who associated COPD or type 2 DM, which makes us cautiously interpret the higher odds of patients with COPD or type 2 DM to present coinfection. Secondly, the retrospective nature of this study precludes a direct assessment of causality between coinfection and specific outcomes.

Future research should focus on larger, prospective cohort studies to further investigate the mechanisms driving the immune response in coinfected patients. Additionally, longitudinal studies could provide insight into the long-term outcomes of patients with extraPTB and COVID-19, particularly regarding the persistence of inflammatory markers and the risk of relapse or complications as well as of long COVID.

This is the first study to comprehensively investigate the unique clinical and paraclinical parameters associated specifically with coinfected patients with extrapulmonary tuberculosis and COVID-19. While numerous studies have examined pulmonary TB or combined pulmonary and extrapulmonary TB in the context of COVID-19, very few have specifically focused on extraPTB alone. This distinct approach allows for a more detailed understanding of the interplay between extraPTB and COVID-19, which has been largely unexplored.

This study provides valuable insights into a unique patient population, potentially paving the way for more targeted clinical strategies and management guidelines for coinfections involving extraPTB and COVID-19.

Furthermore, these findings emphasize the need for integrated TB–COVID-19 management strategies, particularly in resource-limited settings where dual-burden diseases are prevalent. Clinicians should prioritize monitoring IL-6 and PCT levels for early detection of systemic inflammation and prolonged hospitalization risks. Tailored protocols, including targeted anti-inflammatory therapy and vigilant respiratory support, are critical.

Public health policies should incorporate coinfection screening protocols, especially for younger populations presenting with systemic inflammation. Further, the findings underscore the importance of maintaining uninterrupted TB care services.

## 5. Conclusions

This study highlights important clinical and paraclinical differences between patients with extraPTB and SARS-CoV-2 coinfection and those with SARS-CoV-2 infection alone.

The susceptibility of younger individuals to extraPTB underscores the complexity of their immune response. While their heightened immune activity may localize Mycobacterium tuberculosis to extrapulmonary sites, coinfection with SARS-CoV-2 seems to amplify systemic inflammation without severely compromising pulmonary function. This duality highlights the need for targeted management strategies in younger coinfected patients, focusing on controlling systemic inflammation without dampening the antiviral response.

Coinfected patients displayed lower CT involvement scores and higher SpO2 levels, indicating less pulmonary compromise but heightened systemic inflammation, evidenced by elevated IL-6, PCT, and inflammatory ratios. This pattern suggests that extraPTB primarily exacerbates systemic inflammatory responses without worsening pulmonary outcomes compared to SARS-CoV-2 infection alone.

Coinfected patients experienced significantly longer hospital stays, but mortality rates were notably lower compared to the SARS-CoV-2-only group. The lower mortality among coinfected patients might be attributed to their younger age and better-preserved respiratory function despite high systemic inflammation levels. For all patients, lower SpO2 and higher IL-6 levels emerged as independent predictors of mortality, emphasizing the importance of monitoring these parameters closely to identify high-risk cases.

Coinfection with SARS-CoV-2 and extraPTB presents distinct clinical characteristics that differ from SARS-CoV-2 alone, primarily due to heightened systemic inflammation rather than increased pulmonary severity. These insights highlight the need for tailored management protocols that address both inflammatory and respiratory factors, especially for younger and at-risk patients.

These findings underscore the need for tailored treatment and monitoring strategies in coinfected patients to address both the inflammatory and infectious aspects of their disease.

## Figures and Tables

**Table 1:** The number of subjects by sex and age.

		Coinfection Group n = 23	SARS-CoV-2 Group n = 100
Sex	F	10 (43.5%)	48
	M	13 (56.5%)	52
Age	19–40 y	15 (65.2%)	8
	41–65 y	6 (26.1%)	53
	>66 y	2 (8.7%)	36

**Table 2:** Mann–Whitney comparative tests of multiple biological parameters between coinfected and SARS-CoV-2 infected subjects.

Parameter	Coinfection Group Median Value	SARS-CoV-2 Group Median Value	*p*
Age	36	56.5	**<0.001**
BMI	24.45	24.74	0.83
SpO2 at diagnosis	96	94	**0.001**
Lowest SpO2	92	89.5	**0.01**
Peripheral SBP at diagnosis	129	133	0.46
Peripheral DBP at diagnosis	85	87	0.52
CRP	66.1	55.5	0.36
LDH	203	243.5	0.3
IL-6	8.8	1.2	**0.0008**
AST	38	29	**0.005**
ALT	42	30	**0.003**
D-dimer	1.65	0.91	**0.01**
Neutrophils *	6194.8 (SD = 1837.6)	5878.3 (SD = 1162.7)	0.29
Lymphocytes	1460	3010	**<0.001**
Thrombocytes	351,000	252,000	**<0.001**
NLR	3.73	1.83	**<0.0001**
PLR	235.54	77.95	**<0.0001**
SII	1,255,888.88	459,784	**<0.0001**
Chest CT involvement score	6	12	**0.0001**

* The T-student test was employed for neutrophil count. The bolded data in this table represent statistically significant results.

**Table 3:** Anamnestic data as predictors of extraPTB–SARS-CoV-2 coinfection (logistic regression).

Parameter	Odds Ratio	95% CI	Coefficient	Std. Err.	*p*
Age	0.87	0.82–0.93	−0.13	0.03	**0.0001**
Unemployment	0.25	0.08–0.75	−1.36	0.55	**0.01**
COPD	6.5	1.12–37.71	1.87	0.89	**0.03**
Type 2 DM	4.3	1.1–39.66	1.67	0.76	**0.001**

The bolded data in this table represent statistically significant results.

**Table 4:** Paraclinical parameters as predictors of extraPTB–SARS-CoV-2 coinfection (logistic regression).

Parameter	Odds Ratio	95% CI	Coefficient	Std. Err.	*p*
SpO2 at diagnosis	5.37	1.69–17.02	1.68	0.58	**0.004**
PCT	6.5	2.69–10.14	1.1	0.1	**0.01**
CT involvement score	0.49	0.3–0.8	−0.69	0.24	**0.004**

The bolded data in this table represent statistically significant results.

**Table 5:** AUC–ROC analysis for the clinical and anamnestic parameters as discriminators between the presence and the absence of PTB coinfection in SARS-CoV-2 patients (n = 132).

Parameter	AUC	*p*	Associated Criterion	Se%	95% CI	Sp%	95% CI	PPV %	NPV %
Age	**0.81**	**<0.0001**	**<38**	65.22	42.7–83.6	97	91.5–99.4	83.3	92.4
Sex	0.52	0.69	≤1 ^a^	56.52	34.5–76.8	48	37.9–58.2	20	82.8
BMI	0.51	0.85	>18	86.96	66.4–97.2	1	0.03–5.4	16.8	25
Employment status	**0.74**	**0.0003**	**≤1 ^b^**	60.87	38.5–80.3	90	82.4–95.1	58.3	90.9
Smoking status	0.57	0.23	≥1 ^c^	47.83	26.8–69.4	66	55.8–75.2	24.4	84.6
Associated COPD	0.5	0.85	0 ^d^	82.61	61.2–95.0	19	11.8–28.1	19	82.6
Associated type 2 DM	0.53	0.46	0 ^e^	86.96	66.4–97.2	19	11.8–28.1	19.8	86.4
SpO2 at diagnosis	**0.71**	**0.0006**	**>95**	60.87	38.5–80.3	77	67.5–84.8	37.8	89.5
Lowest SpO2	0.66	0.02	>92	47.83	26.8–69.4	80	70.8–87.3	35.5	87
Symptoms severity	0.53	0.39	≤0 ^f^	86.96	66.4–97.2	20	12.7–29.2	20	87
CRP	0.56	0.36	>80	43.48	23.2–65.5	74	64.3–82.3	27.8	85.1
PCT	**0.66**	**0.004**	**>0 ^g^**	47.83	26.8–69.4	84	75.3–90.6	40.7	87.5
LDH	0.56	0.23	≤321	95.65	78.1–99.9	36	26.6–46.2	25.6	97.3
IL-6	**0.72**	**<0.0001**	**>3.6**	78.26	56.3–92.5	69	59.0–77.9	36.7	93.2
AST	**0.69**	**0.007**	**>41**	52.17	30.6–73.2	87	78.8–92.9	48	88.8
ALT	**0.68**	**0.01**	**>44**	47.83	26.8–69.4	97	91.5–99.4	78.6	89
D-dimer	**0.66**	**0.01**	**>1.1**	73.91	51.6–89.8	58	47.7–67.8	28.8	90.6
Neutrophil count	**0.53**	**0.71**	**>7710**	30.43	13.2–52.9	96	90.1–98.9	63.6	85.7
Lymphocyte count	**0.82**	**<0.0001**	**≤2240**	91.3	72.0–98.9	75	65.3–83.1	45.7	97.4
Platelet count	**0.77**	**<0.0001**	**>285,000**	82.61	61.2–95.0	66	55.8–75.2	35.8	94.3
NLR	**0.78**	**<0.0001**	**>2.6**	86.96	66.4–97.2	73	63.2–81.4	42.6	96.1
PLR	**0.86**	**<0.0001**	**>142.63**	91.3	72.0–98.9	80	70.8–87.3	50	94.3
SII	**0.83**	**<0.0001**	**>946,666.66**	86.96	66.4–97.2	79	69.7–86.5	48.8	96.3
Chest CT involvement score	**0.76**	**0.0007**	**≤6**	60.87	38.5–80.3	98	93.0–99.8	87.5	91.6

The bolded data in this table represent statistically significant results. ^a^ (1 = M, 2 = F); ^b^ (1 = employed, 2 = retired, 3 = unemployed); ^c^ (0 = non-smoker, 1 = smoker); ^d^ (0 = no COPD, 1 = associated COPD); ^e^ (0 = no type 2 DM, 1 = associated type 2 DM); ^f^ (0 = non-severe symptoms, 1 = severe symptoms); ^g^ (0 = normal PCT, 1 = elevated PCT).

**Table 6:** Fisher’s exact test: association between several parameters and the likelihood of coinfection.

	Parameter	Fisher’s Exact Test *p*
Presence of extraPTB	Smoking	0.23
	COPD	**0.02**
	Type 2 DM	**0.01**
	Severe symptoms	0.56
	PCT	**0.001**
	Outcome	0.19

The bolded data in this table represent statistically significant results.

**Table 7:** Logistic regression—predictors of fatal outcomes.

Parameter	Odds Ratio	95% CI	Coefficient	Std. Err.	Constant	*p*
Lowest SpO2	0.61	0.46–0.82	−0.48	0.14	38.3	**0.001**
IL-6	1.07	1.01–1.14	0.03	6.35	38.3	**0.01**

The bolded data in this table represent statistically significant results.

## Data Availability

The findings of this study are supported by data available upon request from the corresponding author, though ethical restrictions prevent public access to the data.

## Abbreviations

ALT	Alanine aminotransferase
AST	Aspartate aminotransferase
AUC	Area under the receiver operating characteristic curve
BMI	Body mass index
COPD	Chronic obstructive pulmonary disease
CRP	C-reactive protein
CT	Computer tomography
DM	Diabetes mellitus
HIV	Human Deficiency Virus
IL-6	Interleukin 6
LDH	Lactate dehydrogenase
*n*	Number of subjects
NK cells	Natural killer cells
MTB	Mycobacterium tuberculosis
OR	Odds ratio
*p*	*p*-value
PCT	Procalcitonin
PTB	Pulmonary tuberculosis
ROC	Receiver operating characteristic
RT-PCR	Reverse transcription polymerase chain reaction
SpO2	Saturation of peripheral oxygen
TB	Tuberculosis
WHO	World Health Organization
